# Analysis of Tracheobronchial Diverticula Based on Semantic Segmentation of CT Images *via* the Dual-Channel Attention Network

**DOI:** 10.3389/fpubh.2021.813717

**Published:** 2022-01-05

**Authors:** Maoyi Zhang, Changqing Ding, Shuli Guo

**Affiliations:** ^1^School of Control Science and Engineering, Beijing Institute of Technology, Beijing, China; ^2^Department of Imaging, Fengxian People's Hospital, Xuzhou, China

**Keywords:** tracheobronchial diverticula identification, semantic segmentation, deep learning, dual-channel, attention

## Abstract

Tracheobronchial diverticula (TD) is a common cystic lesion that can be easily neglected; hence accurate and rapid identification is critical for later diagnosis. There is a strong need to automate this diagnostic process because traditional manual observations are time-consuming and laborious. However, most studies have only focused on the case report or listed the relationship between the disease and other physiological indicators, but a few have adopted advanced technologies such as deep learning for automated identification and diagnosis. To fill this gap, this study interpreted TD recognition as semantic segmentation and proposed a novel attention-based network for TD semantic segmentation. Since the area of TD lesion is small and similar to surrounding organs, we designed the atrous spatial pyramid pooling (ASPP) and attention mechanisms, which can efficiently complete the segmentation of TD with robust results. The proposed attention model can selectively gather features from different branches according to the amount of information they contain. Besides, to the best of our knowledge, no public research data is available yet. For efficient network training, we constructed a data set containing 218 TD and related ground truth (GT). We evaluated different models based on the proposed data set, among which the highest MIOU can reach 0.92. The experiments show that our model can outperform state-of-the-art methods, indicating that the deep learning method has great potential for TD recognition.

## Introduction

### Background

Tracheobronchial diverticula (TD), or paratracheal air cystic (PTAC), is defined as a congenital or acquired air cyst located in a lateral wall of trachea (1). Specifically, it is a cystic lesion involving primarily the outpouching of the trachea and main bronchial lumen and is located mainly on small round air cysts lesion beside the superior mediastinal trachea.

Tracheobronchial diverticula, mostly discovered in the chest CT by accident, is usually asymptomatic. Chronic cough, dyspnea, stridor or recurrent tracheitis, dysphagia, dysphasia, odynophagia, neck pain, hoarseness, suffocation, and hemoptysis caused by TD are less frequent. In rare cases, TD may lead to the chronic infection of the tracheobronchus. Only when the relationship between TD and symptoms is confirmed can the symptomatic treatment of antibiotics, mucus solubilizers, and physical therapies be used. The endoscope check includes electrolumes and laser or electroconding endoscopes. Inspections including detachable and longitudinal frame suction and biopsy, thoracoscope, braking, and longitudinal isolation are only needed in rare cases. Before the advent of the CT, due to insufficient understanding of this disease, the lack of specificity of the clinical symptoms, and the restrictions of imaging methods such as chest portions, only severe symptoms could be diagnosed. Thanks to the wide application of multislice spiral CT and the advancement of computer technology, the detection rates of the tracheal and bronchial diverticulums are greatly improved. The coronary and sagittal images are useful in showing the relationship between the tracheal cavity and TD.

**Figure 1:**
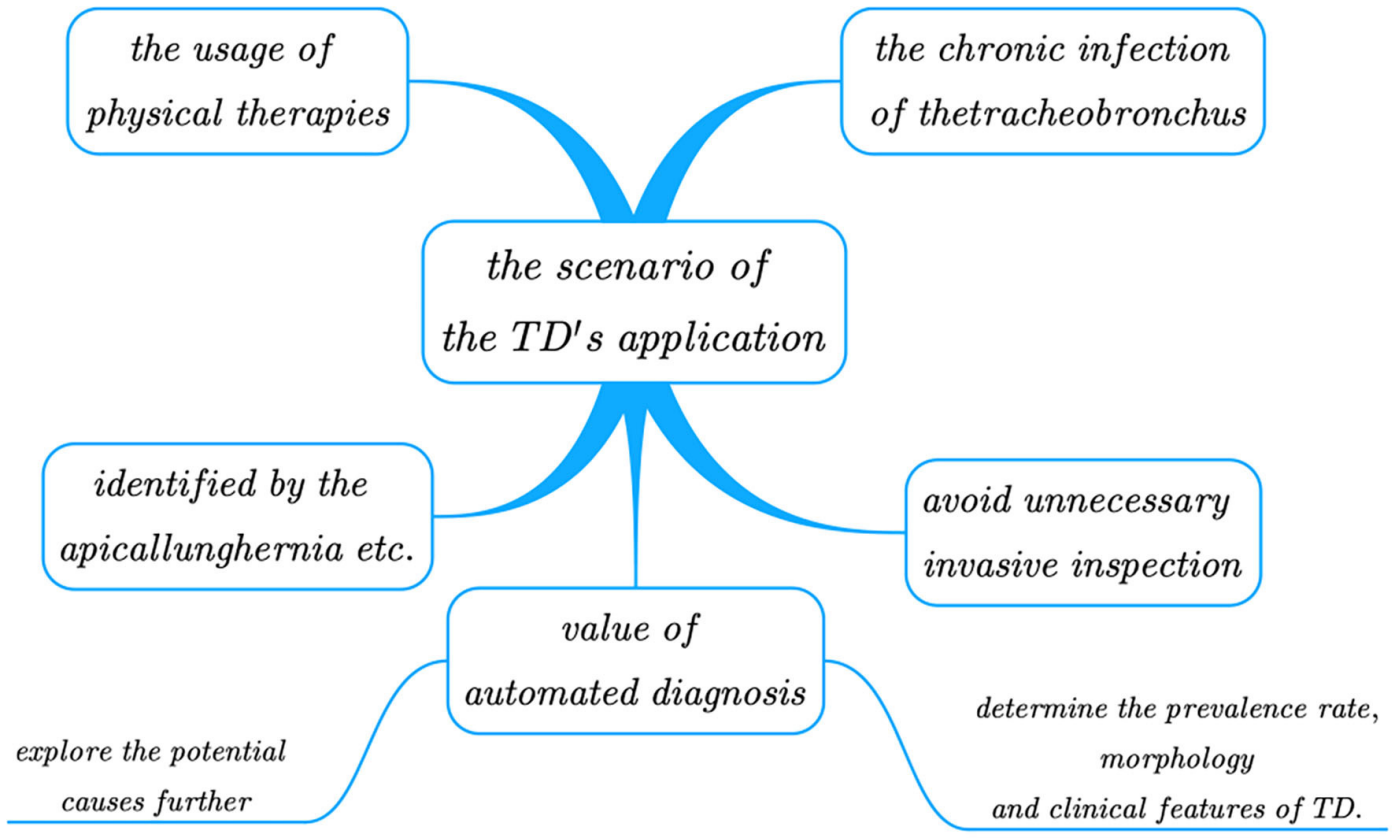
The scenarios of the TD's application.

Tracheobronchial diverticula is mainly identified by the following lesions: apical lung hernia, trachea laceration, paramediastinal emphysema, esophageal diverticulum, paratracheal air cyst, pneumomediastinum, subcutaneous cervical emphysema, mediastinal emphysema, cervical subcutaneous emphysema, and cervical tracheal duplication. CT should be taken as the main diagnostic tool. The whole picture of the diverticulum and its communication with trachea and bronchi can be observed through thin-section CT and its 3D reconstruction; so we should give full play to spiral CT's advantages in diagnosing tracheal diverticulum, and avoid unnecessary invasive inspection. Bronchoscopy is another tool for diagnosis, but with it, some patients suffering from tracheal occlusion can be misdiagnosed as narrow neck TD or fibrous connection. Unfortunately, not all physicians have sufficient understandings in this regard, as a large number of missed diagnoses still occur in many hospitals worldwide. In addition, since the lesion area of TD is small it poses great challenges to diagnosis. Therefore, designing an efficient and accurate automated method to detect TD can significantly reduce the workload and the missed diagnosis or misdiagnosis rates, ultimately enhancing the diagnostic effectiveness. Once the method is widely applied to hospitals, large sets of diagnostic data obtained can be utilized to explore the potential causes further, and to determine the prevalence rate, morphology (i.e., quantity, size), and clinical features of TD. For a clearer illustration, Figure 1 shows the scenarios of TD's application.

### Related Work

The related work is divided into four parts: tracheobronchial diverticula, machine learning, deep learning, and cloud technology.

#### Research Status of TD

Few cases of TD have been reported since the first description by Rokitansky in 1838 (2). With the increasingly wide application of CT as a high-performance and non-destructive testing method in the medical field, especially as the main screening method for early lung cancer, the detection rate of PTAC has increased significantly by about 4–8.1% (3–5), which is 1% higher than previously reported postmortem prevalence (0.3% in children) (6). Pace et al. suggested that this disease was associated with the congenital defect or abnormal development of the posterior tracheal membrane, or possibly with acquired chronic obstructive pulmonary disease (7). Ahmet et al. used chest multidetector computed tomography (MDCT) to determine the prevalence of paratracheal air cysts and their correlation with different lung diseases, and finally proved that air cysts are associated with chronic obstructive pulmonary disease (3).

Unlike traditional medical segmentation, the lesion area of TD is relatively small in both real conditions and CT image. In the medical field, most researchers only reported related cases and gave prescriptions but did not design an automatic method for detection.

#### Research Status of Machine Learning

In the field of machine learning, Deepa et al. (8) used Ridge Adaline Stochastic Gradient Descent Classifier for the early prognosis of diabetes, with higher accuracy than other algorithms. Dhanamjayulu et al. (9) adopted machine learning algorithms to identify malnutrition from facial images. Ghazal et al. reviewed the machine learning in smart healthcare (10) and an improved SVM method for diagnosing Hepatitis C (11). Shah et al. (12) proposed two gender-based risk classification and age-based risk classification methods to identify cardiovascular diseases (CVD). The accuracy of the model can reach 98%, which improved the identification efficiency. In addition, the progress of machine learning in the direction of medical data mining (13) is also gratifying.

#### Research Status of Deep Learning

In recent years, deep learning (14) has shown a booming trend of development, in which many networks for medical segmentation of small and medium targets have emerged. For example, U-net (15) is specially developed for medical image segmentation. Its U-shaped structure is an asymmetrical structure of up- and downsampling, whose feature mapping can be connected through the intermediate jump connection, thus enriching the extracted semantic information. Shrivastava et al. (16) presented a learning mechanism method for cognitive maps, which has been successfully applied to identify problems in targets. Jégou et al. (17) restored the complete input resolution by adding an up-sampling path, and extended DenseNets to FCN to reduce the impact of large computation and parameters, resulting from high-resolution characteristic patterns multiplied by a large number of input filters. However, these networks have difficulties in the segmentation of small and medium targets.

In view of this, researchers combined the detection and segmentation tasks to produce a series of networks. Mask RCNN (18) is the most advanced instance segmentation technology, with which, the pixel-level labels in each prediction box can be predicted, and multitask segmentation can be completed. However, its pooling layer using “ROI-align” reduces the resolution of input features, resulting in a loss of spatial detail of the image. In addition, it will increase computer overheads and network parameters. BlitzNet (19) used a full convolutional network to detect and segment the target and promoted model learning through weight sharing between the two tasks. But the model did not show the advantages of segmenting tasks. Based on the network mentioned above, Zhang et al. (20) introduced an ROI convolution directly in the location network area. This convolution module operates on all ROI during calculation, and it is applied to acetabulum segmentation from ultrasonic images. In addition, it helps solve the segmentation problem of small targets by experimentally demonstrating the introduction of localization units. Bak et al. (21) used the deep learning model to analyze the LDCT images of 131 patients, and calculated related indicators such as the emphysema index and mean lung density. Deep learning and statistical methods were also combined to detect emphysema quantitatively. Jin et al. (22) proposed a two-stage deep learning structure to improve the accuracy of emphysema quantification. Srinivasu et al. (23) proposed an algorithm based on probabilistic deep Q network for real-time robotic surgery, showing excellent performance in terms of the learning rate. Lin et al. (24) developed a deep learning model for COVID-19 identification and compared the CT images of TD and the coronavirus. Deep learning methods are also widely used in face recognition (25), breast cancer detection (26), and cervical cancer classification (27).

#### Research Status of Cloud Technology

With the development of cloud technology and blockchain technology, Ghayvat et al. (28) combined the blockchain (BC)-based confidentiality-privacy (CP) preserving scheme, which improved the accuracy by 12% compared with the traditional algorithm, verifying that it is a good solution for big data storage and privacy protection. At the same time, they also designed a COUNTERACT system that can calculate the incubation period of new crown objects (29) and an environmentally assisted living system (30) that well combines the latest sensor fusion technology. These designs will be the trend of future applications.

### The Deficiency of Existing Research

Although improvements have been made in the performances of various methods based on deep learning, there is still room for breakthroughs in some areas. The motivations for the proposed methods can be summarized in the following two aspects:

First, the existing TD identification task relies mostly on the data collected by physicians in the diagnosis process, most of which needs to be visually observed by professionals and written into the diagnosis report. Since TD lesion sites are not obvious, identification and diagnosis by manual means are prone to fatigue, misdiagnosis, and missed diagnoses. In existing models of lung diseases based on deep learning, most of the focuses are on cancerous sites (31, 32), pulmonary nodules (33–35), etc. Many studies have ignored the effect of paramediastinal air cysts on the subsequent disease.

Secondly, the existing methods are not robust, but in TD semantic segmentation, the identifiable lesion covers only a small area with low pixels, and edge restoration is needed. Moreover, the TD's position in CT image needs to be identified, which can hardly be achieved by applying existing methods to these datasets. The advancement and the limitations of the current study can be concluded in Table 1.

**Table 1:** The conclusion of advancement and the limitations of the current study.

**Advancement**	**Limitations**
TD Case reports	Not robust
Machine learning, with deep learning focused on other areas of interest on regions	Few scholars have automated the diagnosis of TD

### The Novelties and Contributions of the Proposed Work

To address the above limitations, we propose a novel structure to classify every pixel of TD through a dual-channel attention-based segmentation network. The main features of the proposed method are as follows:

1. Unlike the traditional bounding box-based target detection, or the entire image-based classification task, we performed the semantic segmentation on the TD lesions identification by classifying each pixel point by pixel. This method helps to better utilize the original data to present a clearer image, which is convenient for subsequent diagnosis. This proposed method is the first semantic segmentation network for TD diagnosis.
2. The proposed method saves the traditional intermediate steps (feature extraction) by taking the end-to-end loss function as the features of the generated evaluation and the GT confirmation. It can directly and automatically learn the overall lesion characteristics instead of manual identifications that undermine the generalization performance.
3. Given the outstanding performance of the attention mechanism on semantic segmentation tasks, we proposed a novel dual-channel attention TD segmentation network with position coding and contour coding modules, which has better accuracy and robustness. We compared the proposed model with the traditional various semantic segmentation models based on deep learning. The MA and MIOU of our method can be as high as 0.92 and 0.87, respectively. On this basis, we also carried out corresponding ablation experiments to verify the advantages of the added modules.

### Paper Organization

The rest of the paper is organized as follows: first, in the “Experimental Methods and Materials” section, we introduced the data sources and preprocessing methods; also, the proposed model is elaborated in this section. As for the “Results and discussion” section, a series of comparative and ablation experiments are described. Finally, we summarized this work and pointed out the directions for future research in the conclusion section.

## Experimental Methods and Materials

### Experimental Data and Preprocessing

**Figure 2:**
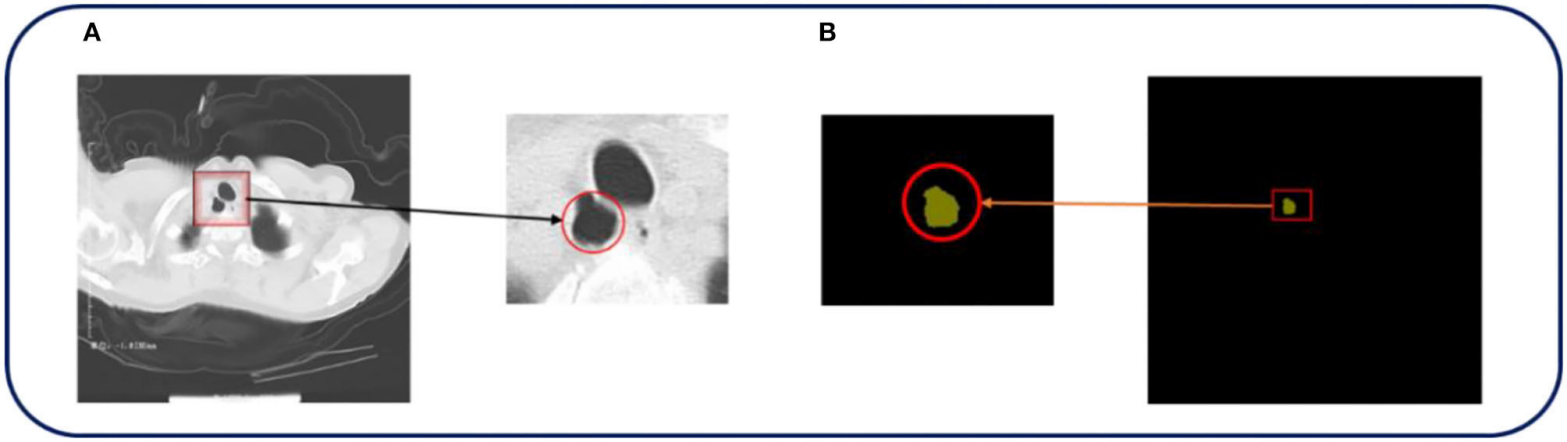
The original CT image and the corresponding ground truth are shown in Figure 1. **(A,B)** In the above pictures, red represents TD, which is the part that we are interested in.

The cases included in this study were patients who underwent routine chest CT scanning using Philips Brilliance 16- and 64-slice spiral CT machine in the Department of Medical Imaging, Fengxian People's Hospital, Jiangsu Province, from October 2020 to January 2021. The patients were scanned by CT from the superior border of the thyroid cartilage to the bottom of the lung. The field of vision was 350 mm, 120 kV, 250 mA, and the collimator width was 0.75 mm. The original data of each period were reconstructed with 1 mm layer thickness and 0.15 mm layer spacing, and postprocessing techniques such as multiplane recombination (MPR), maximum density projection, and volume reconstruction were applied at the workstation. The ground truth we annotated based on the original image was all annotated and verified by experienced radiologists. The image size is 512^*^512, and all data used for training, testing, and verification are desensitized. Figure 2 shows a set of examples.

In the collected pathological images, the lesion area is highly obscure, where light environment or photographing can easily lead to background pollution, so it is challenging to segment the lesion area accurately. Therefore, we mainly used two methods to preprocess the data. Firstly, the data of HSV hue transformation were enhanced, which can avoid the influence of light on the image, making the network more robust and the color space clearer compared with the method of RGB color space. HSV can be described as follows:


(1) $$\begin{array}{r} \text{Output}=\text{HSV}\left({\text{x}}_{\text{i}=\text{r},\text{g},\text{b}}\cdot {\text{α}}_{\text{i}=\text{h},\text{v},\text{s}}\right) \end{array}$$

where, *x* is the single channel of input image (*i* = *r, g, bi* = *r, g, b*); *a* (*j* = *h, s, vj* = *h, s, v*) is the disturbance factor; HSV is the augmented model of color space. In our experiment, *h* = 0.015, *s* = 0.7, *v* = 0.4*h* = 0.015, *s* = 0.7, *v* = 0.4.

Secondly, we adopted the nonlinear signal processing technique based on ranking statistical theory to suppress noise for effective preprocessing. Specifically, in the small lesion area, it is more prominent with the effect of salt and pepper noise. Therefore, we created a sliding window near the lesion area, which traverses all pixels of the area, accumulates all pixel values, and takes the median one as the enhanced pixel. It can effectively protect the edge information of the image while suppressing noise points. The equation can be expressed as:


(2) $$\begin{array}{r} \text{h}\left(\alpha ,\beta \right)=\text{ med}\left\{f\left(\text{α}-m,\text{β}-n\right),m,n\;\in W\right\} \end{array}$$

where *f*(α, β) and *h*(α, β) denote the original image and the processed image, respectively, and W is the two-dimensional input template. *med* represents the convolutional implementation of the sliding window. All the original images used for network design were 512 × 512 pixel in size, with a horizontal and vertical resolution of 96 dpi. The image size was kept the same to accommodate the CNN network training.

### Network Structure

#### Overall Structure

**Figure 3:**
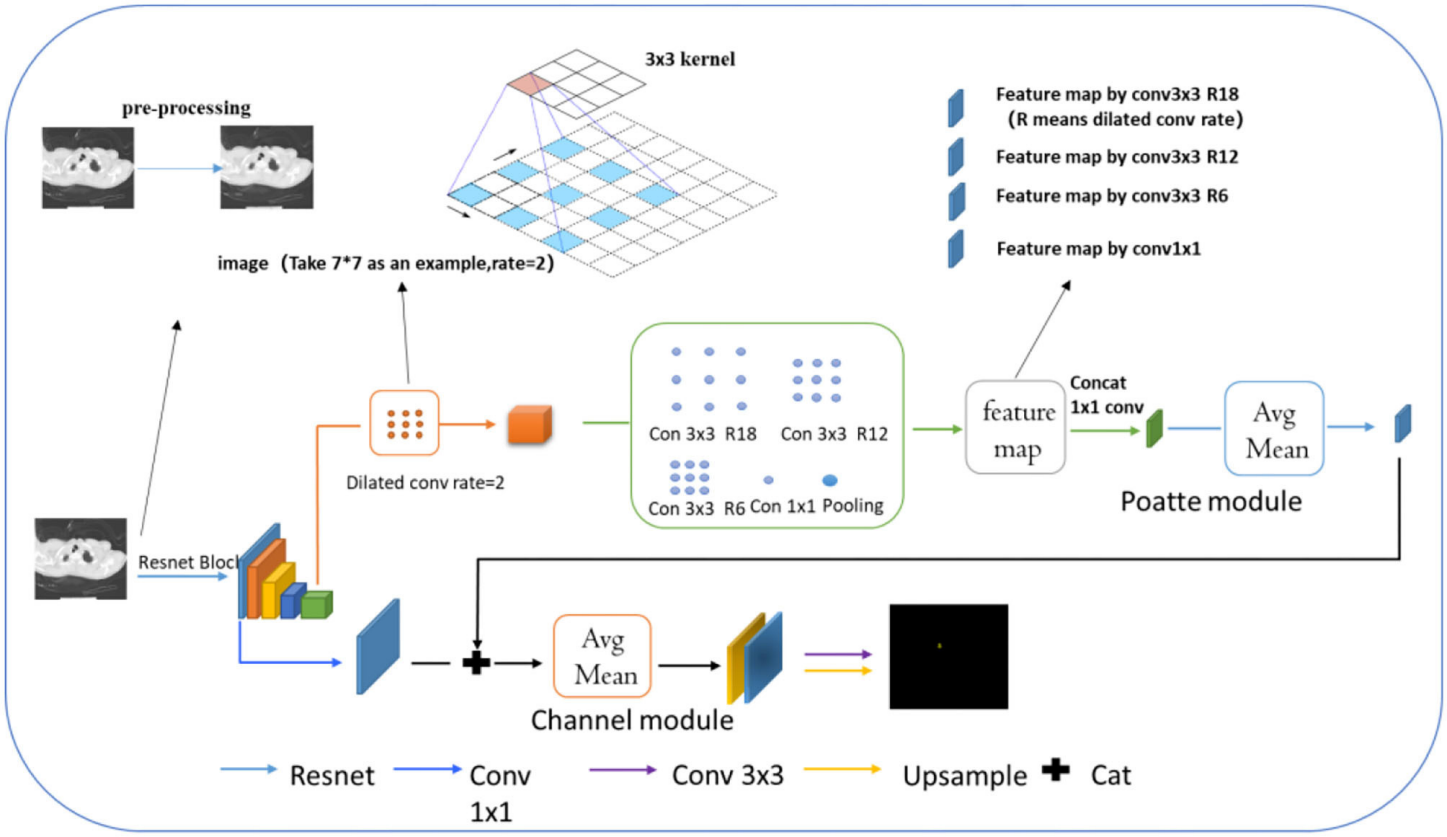
Attention-based tracheobronchial diverticulum segmentation network.

This part mainly introduced the network based on attentional tracheobronchial diverticulum segmentation, as shown in Figure 3. Firstly, the resnet101 network was employed as the backbone to extract the deep and shallow features of the image. In addition, considering the smallness of the lesion and its complex background, a two-channel network was designed to achieve the segmentation task with both rough and fine-grained results, which consisted of two parts—one was location feature extraction and encoding module, which extracts multiscale spatial features of the object to be segmented and locating the target area in the image, and the other was the contour encoding module, which was in charge of fusing and upsampling (mapping) the output of the initial Conv-2 feature of Resnet101 and the location encoding module, so as to further enhance abilities of details recovery of contour edges of the image.

Further explanation about the model is made in the following. Before extracting features by Resnet, we used the preprocessing model mentioned above to process the TD image. Dilated convolution is a common one whose working mode is explained in the image by arrows. Compared with conventional convolutions, its receptive field offers more lesion information. Then, different feature maps by various types of dilated convolution can be received, which are processed by concat in the next step.

#### Location Encoding Module (Poatte Module)

Using Resnet101, we extracted features of deep and shallow layers. In the location encoding module, the target area should be first located in the image, around the left hand and close to the surrounding trachea and the right upper lung tip. Then we employed the deep layer to extract the trachea and right upper lung tip features and further identified the esophagus area. However, in this layer, smaller targets tended to disappear after multiple convolution pooling. For that, the dilation (ASPP) convolution (36) (expansion rate of 2) was used to increase the receptive field of the image and decrease the probability of the disappearance of small targets in the deep feature map. In the case of two-dimensional signals, for each position *i* and *a* convolution filter *w* on the output feature map *y*, they were applied by atrous convolution on the input feature map *x*, as follows.


(3) $$\begin{array}{r} \text{y}\left[\text{i}\right]=\underset{\text{k}}{\sum }\text{x }\left[\text{i}+\text{r}\cdot \text{k}\right]\text{ w }\left[\text{k}\right] \end{array}$$

In the above function, the atrous rate *r* determined the stride, with which we sample the input signal, and the output y of ASPP through the position module was:


(4) $$\begin{array}{rcl} \text{Ms }\left(\text{F}\right) & = & \text{σ }\left(\text{f }\left(\left[\text{AvgPool }\left(\text{F}\right);\text{MaxPool }\left(\text{F}\right)\right]\right)\right) \end{array}$$

(5) $$\begin{array}{rcl} \text{Ms }\left(\text{F}\right) & = & \text{σ }\left(\text{f}1\times 1\;\left(\left[\text{Fs avg };\text{ Fs max}\right]\right)\right) \end{array}$$

In the formula, f1 × 1 was the convolution kernel of 1 × 1, and σ was the sigmoid function.

**Figure 4:**
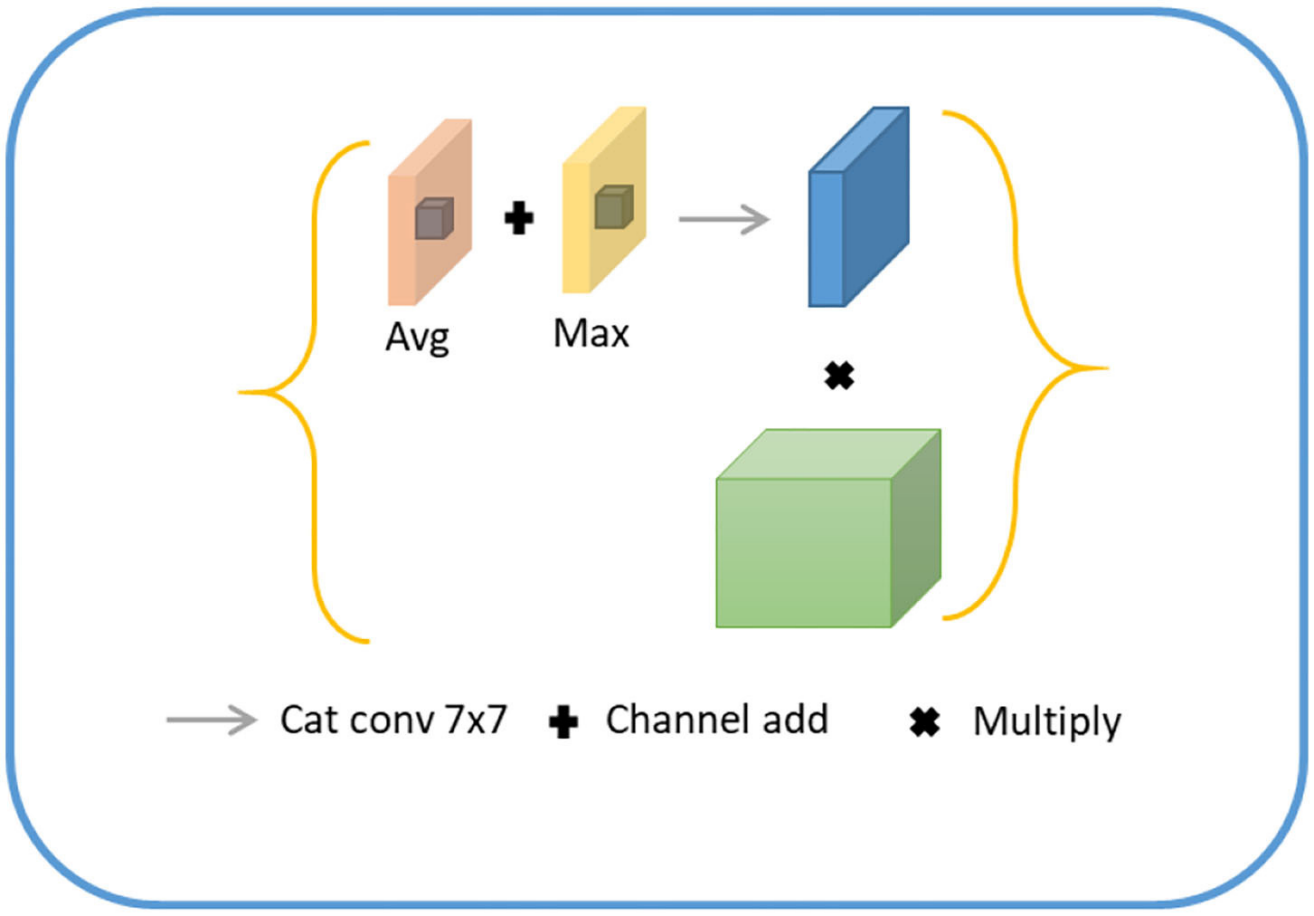
Schematic diagram of location coding module.

Besides, the chosen single deep scale was conducive to large target detection but poor for the small one. On this single scale, what 3 × 3 convolution and 1 × 1 convolution can capture was only local region features rather than the global ones. Therefore, the feature map obtained by the upper expansion convolution was conducted by different expansion rates. With the increase of convolution layers, the convolution kernel degenerated into 1 × 1 when the expansion rate of convolution was large, which could not capture global ones. To solve the problem caused, we first added image-level features, specifically, the average pooling of each channel of the feature map, and then the original scale resolution was sampled up. Secondly, we set the expansion rates of these convolutions to 6, 12, 18, and ultimately fused the average pooling features with the features of different expansion rates. The fused features possessed characteristics of global context so that the target at different scales can be roughly located. Meanwhile, because the ultimate goal was to find small lesion areas around the large target esophagus, fused features were put into the location attention module, as shown in Figure 4. The module first pooled the input features into global maximum and global average pooling and fused them into 7 × 7 convolutions, which were positionally expressed, and this generally made the target location more salient. Finally, the information of location was multiplied by the characteristics of the previous layer to further identify the small target position.

#### Contour Coding Module (Channel Module)

**Figure 5:**
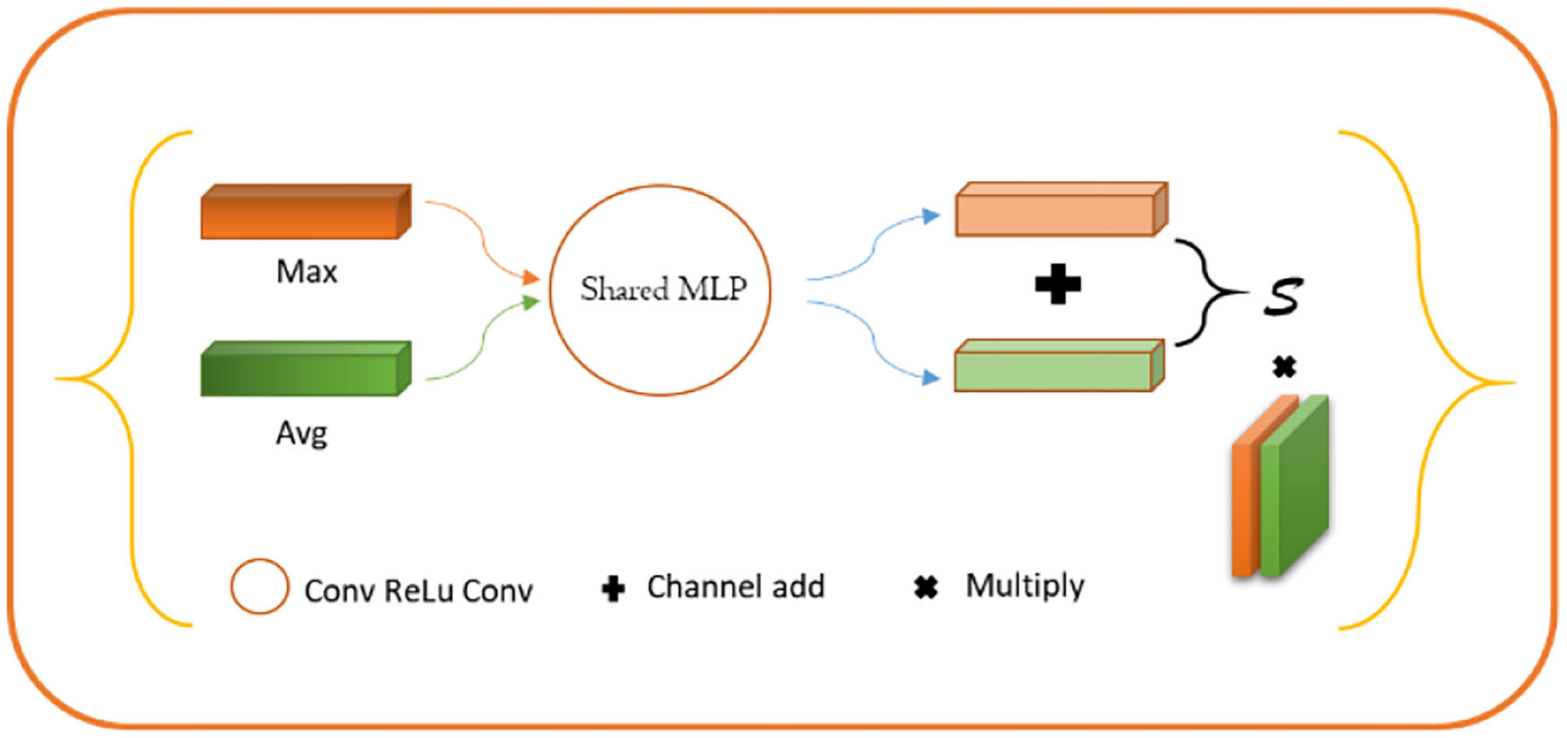
Schematic diagram of contour coding module.

For lesion being a small object, the spatial information of the target was usually lost after deep convolution so that shallow features were employed to restore the contour information of the target. Specifically, we first conducted a 1 × 1 convolution of shallow features, fused with the input of the position-coding module to endow them with the contour information of shallow features and position of deep features. Next, the fused feature was processed through the attention module of channel fusion, as shown in Figure 5, where two 1 × 1 × C feature maps were obtained by planar and maximum pooling of the fused features in terms of the long and wide directions. The final channel feature was strengthened by a shared convolution layer and pixel-level sum operation, making the initial feature profile the target area. Finally, the feature map was recovered to the original scale size by convolution and upsampling.

#### Experiment Settings

**Figure 6:**
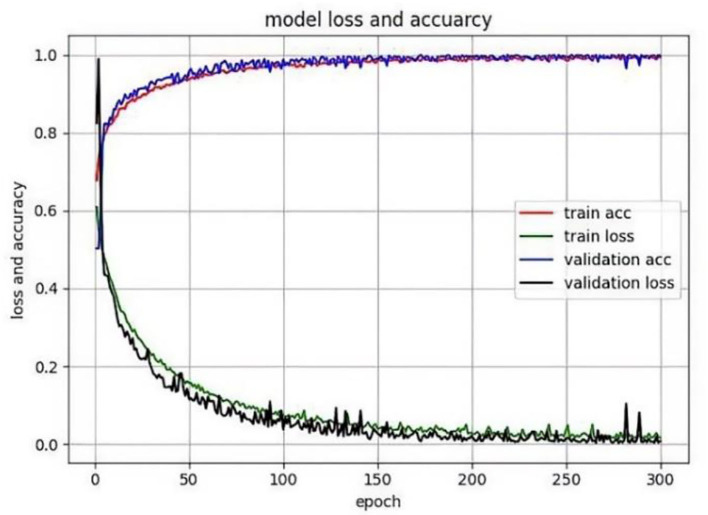
Model training chart.

The method was implemented based on the Pytorch library and ubantu environment, with the learning rate being 0.0001, the momentum and weight decay coefficients 0.9 and 0.0001, respectively. The model was trained by GTX 2080 Ti, Batchsize = 4, and the training epoch was 300. When the epoch was 0 to 150, the model converged quickly, improving accuracy. But it was relatively stable and converged moderately with slow-moving accuracy between 150 and 300, as shown in Figure 6. In addition, the TD lesion dataset was obtained from the hospital, and 218 images (190 images in training set, 10 images in validation set, and 18 images in test set) and 218 lesion area markers were constructed under the guidance of pathologists.

Based on this, the pretraining model with fine adjustment is applicable in a resource-constrained environment. The resources consumed are only the computational ones that are returned in the following gradient. Moreover, the following multiscale module of feature extraction and feature attention do not require a great number of computing resources. In the multiscale feature extraction module, we used dilated convolution to expand the receptive field and decrease the representation of feature maps, and even the demand for resources. We repeatedly utilized 1^*^1 convolution in the module to compress features to integrate representation of features and lower demands for resources. Based on the above two points, the model can be trained and tested in a resource-constrained environment.

### Evaluation Indexes

We adopted two most essential evaluation indexes of semantic segmentation, namely, mean pixel-level accuracy (MPA) and mean intersection over union (MIoU), which were as follows :


(6) $$\text{MPA }=\frac{\text{1}}{\text{T}}\sum_{\text{T=1}}^{\text{T}}\;\left[\frac{\text{1}}{\text{C+1}}\sum_{\text{m=0}}^{\text{C}}\frac{{\text{p}}^{(\text{mm)}}}{\sum_{\text{n=0}}^{\text{C}}{\text{p}}^{(\text{nn)}}}\right]$$

(7) $$\begin{array}{l} \text{MIoU}\\ \;=\frac{\text{1}}{\text{T}}\sum_{\text{T=1}}^{\text{T}}\;\left[\frac{\text{1}}{\text{C+1}}\sum_{\text{m=0}}^{\text{C}}\frac{{\text{p}}^{\left(\text{mm}\right)}}{\sum_{\text{n=0}}^{\text{C}}{\text{ p}}^{\left(\text{mn}\right)}\text{ + }\sum_{\text{n=0}}^{\text{C}}{\text{ p}}^{\left(\text{nm}\right)}{\text{ − p}}^{\left(\text{mm}\right)}}\right] \end{array}$$

In the above functions, *p*^(*mn*)^ denotes the true value of m, predicting the correct number of TD lesions. *C* + 1 was the number of categories (including empty classes). *T* was the number of CT images of the tracheobronchial diverticulum, which was 218 in this study. *MPA* was the average value of the correct classification of calculating pixel categories, *MIoU* was generally calculated based on the categories, that is, the *IoU* of each category was accumulated after the calculation, and then their average was the holistic evaluation.

## Results and Discussion

### Comparison With Advanced Semantic Segmentation Algorithms

In this part, we selected the classic and novel semantic segmentation networks, including FCN, DENSE, DANet, PSPNet, and DoubleUnet. Among these networks, FCN (37) was the first network that applied CNN structure to semantic segmentation tasks and altered many classification networks into fully convolutional networks. Then, by introducing Atrous convolution, DENSE solved the coding problem of multiscale information and obtained the spatial pyramid structure suitable for semantic segmentation. DANet (38) proposed a dual attention network for segmentation, which could introduce spatial and positional attention to adjust the local semantic information in the adaptive integrated image. PSPNet (39) proposed a pyramid network that could extract context semantic information, improving the segmentation performance in complex scenes. DoubleUnet (40) employed two stacked U-Net (one pretrained coding network was used to capture more abundant semantic information, and the other was set at the bottom to extract information) to improve the segmentation performance of multiple sets of medical images.

To verify the effectiveness of our dual-channel network, MPA and MIOU were adopted as evaluation indexes to compare (SOTA) FCN32S, FCN16S, FCN8S, FCN, DENSE, DANet, PSPNet, DoubleUnet network, and build different algorithms. We selected VGG-16 (41) and ResNet-50 (42) as feature extraction networks, respectively. The former obtained more semantic information by deepening the network depth. The latter solved the problem of gradient explosion and disappearance by introducing residual structure and enhancing the generalization performance of the network with wide applications in classification and segmentation tasks in many scenarios. We conducted the experiment combining the above backbones with the segmentation network, and the results are shown in Table 2. It can be seen from Table 1 that the MIOU value of FCN network, after establishing VGG16 and ResNet50, was about 0.4–0.5, the figure after DENASPP121,161,169,201 as about 0.6–0.7, the one after ResNet series network was about 0.5–0.6, the figure after ResNet series network was approximately 0.3, and the accuracy of DoubelUnet network was about 0.53. However, the MIOU accuracy of the proposed network was 0.87, much higher than the currently popular algorithms, mainly due to the effective extraction of spatial features and the efficient use of boundary information in the network.

**Table 2:** Results of common semantic segmentation methods in identifying tracheobronchial diverticula.

**Method**	**Backbone**	**MPA**	**MIoU**
FCN32S	VGG16	0.77	0.50
FCN16S	VGG16	0.74	0.49
FCN8S	VGG16	0.75	0.49
FCN	ResNet50	0.78	0.59
FCN	ResNet50	0.81	0.53
DENSE	DENASPP121	0.87	0.64
DENSE	DENASPP161	0.89	0.78
DENSE	DENASPP169	0.78	0.62
DENSE	DENASPP201	0.78	0.63
DANet	ResNet50	0.77	0.55
DANet	ResNet101	0.77	0.58
DANet	ResNet152	0.88	0.63
PSPNet	ResNet50	0.60	0.34
PSPNet	ResNet101	0.63	0.34
PSPNet	ResNet152	0.63	0.34
DoubleUnet	ResNet50	0.78	0.53
**Ours**	ResNet101	**0.92**	**0.87**

*The meaning of the bold values is that the network we designed in this article (We call it ours for short)*.

In addition, Resnet50, Mobilnetv2 (43), Drn16 (44), and Xcepotionv2 (45) were also selected as backbones for the dual-channel network. As for their results, the highest MIOU was 0.87 and the lowest was 0.77, both lower than the proposed algorithm, showing the robustness and efficiency of the proposed network. The comparison is shown in Table 3.

**Table 3:** Comparing the results of our model with different backbones for identifying tracheobronchial diverticula.

**Method**	**Backbone**	**MPA**	**MIoU**
Ours	Resnet50	0.90	0.87
Ours	Mobilenetv2	0.90	0.83
Ours	Drn16	0.92	0.87
Ours	Xceptionv2	0.84	0.77

First of all, we compared the indexes of different backbones, and their good performance proved the universality and validity of the segmentation of modules. ASPP module was proposed for the multiscale problem of objects. Before the convolution, it was resampled at a given feature layer with multiple sampling rates. We used multiple paralleled dilated convolution layers with different sampling rates to expand the receptive field and enhance the expression of features. In the model, the convolution with a convolution kernel of 1 was used repeatedly to compress, integrate, and increase nonlinearity. In view of the obscure feature points of the object, we adopted the global average pooling to enhance the response in the channel further, making the feature more prominent and conducive to the segmentation. Channel attention is to strengthen the learning of different fields of vision features. Furthermore, spatial attention is to enhance the learning of spatial expression features of objects and the response of feature points. Based on the above points, the model we designed strengthens feature learning, thereby improving segmentation accuracy.

**Figure 7:**
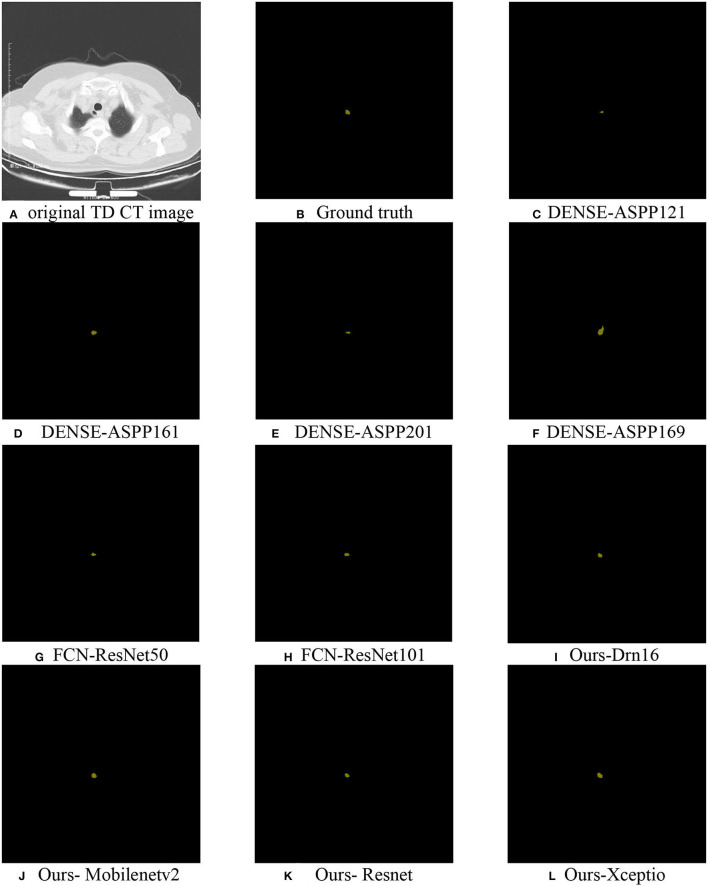
TD lesion segmentation diagram: **(A)** the original CT data; **(B)** ground truth; **(C–H)** partially selected comparison algorithm; **(I–L)** predicted results using Drn16, Mobilenetv2, Resnet, and Xception as the encoder extraction network, respectively.

For a more direct exhibition of the algorithm, we selected algorithms used for the above comparison and the TD lesion segmentation map (Figure 7) by the improved algorithm, which was compared with ground truth.

In the experiment, we found that the recognition results of popular algorithms (such as DoubleUnet) were overfitting, causing the incomplete identification of the lesion area for its smallness and distraction of organs around it. However, the network was designed by setting a position-coding module and a contour-coding module, introducing positional and spatial attention to focus on extracting the semantic information of the lesion area, which made its segmentation effect better than the existing algorithms.

### Ablation Experiment

Atrous convolution and positional attention module, and spatial attention module (PCM) were added to the network. To verify the effectiveness of the two modules, we performed the following ablation experiments in Table 4. When there was no module in the network, the evaluation index MIOU was 0.68. When deep and separable convolution was added, it was 0.79, and the accuracy value was higher than that of the original network 0.11. The reason was that it selected convolutions with different expansion rates when extracting features at high scales to increase the receptive field, making features more holistic and avoiding the loss of target information. When positional and spatial attention modules (PCM) were added, the MIOU accuracy was 0.82 without dilated convolution, higher than 0.14 of the original network. The reason behind it was that the two attentional modules made the target position prominent. Combining the deep and separable convolution (convolution with different expansion rates, Atrous convolution), positional attention module, and spatial attention module (PCM) with the network, the accuracy was 0.87, higher than the original accuracy of 0.68. The ablation experiment further verified the effectiveness of the modules.

**Table 4:** Comparison of the results of our model with different backbones for identifying tracheobronchial diverticula.

**Atrous convolution**	**PCM**	**CAM**	**MIoU**
×	×	×	0.68
√	×	×	0.79
×	√	√	0.82
√	√	√	0.87

Furthermore, combined with the visualized results of the above segmentation, it can be clearly found that the proposed algorithm can effectively locate and segment TD lesions. Meanwhile, the addition of attentional modules improved performance, especially reducing the false detection of small objects and providing richer positional, spatial, and background information than the ordinary network.

**Figure 8:**
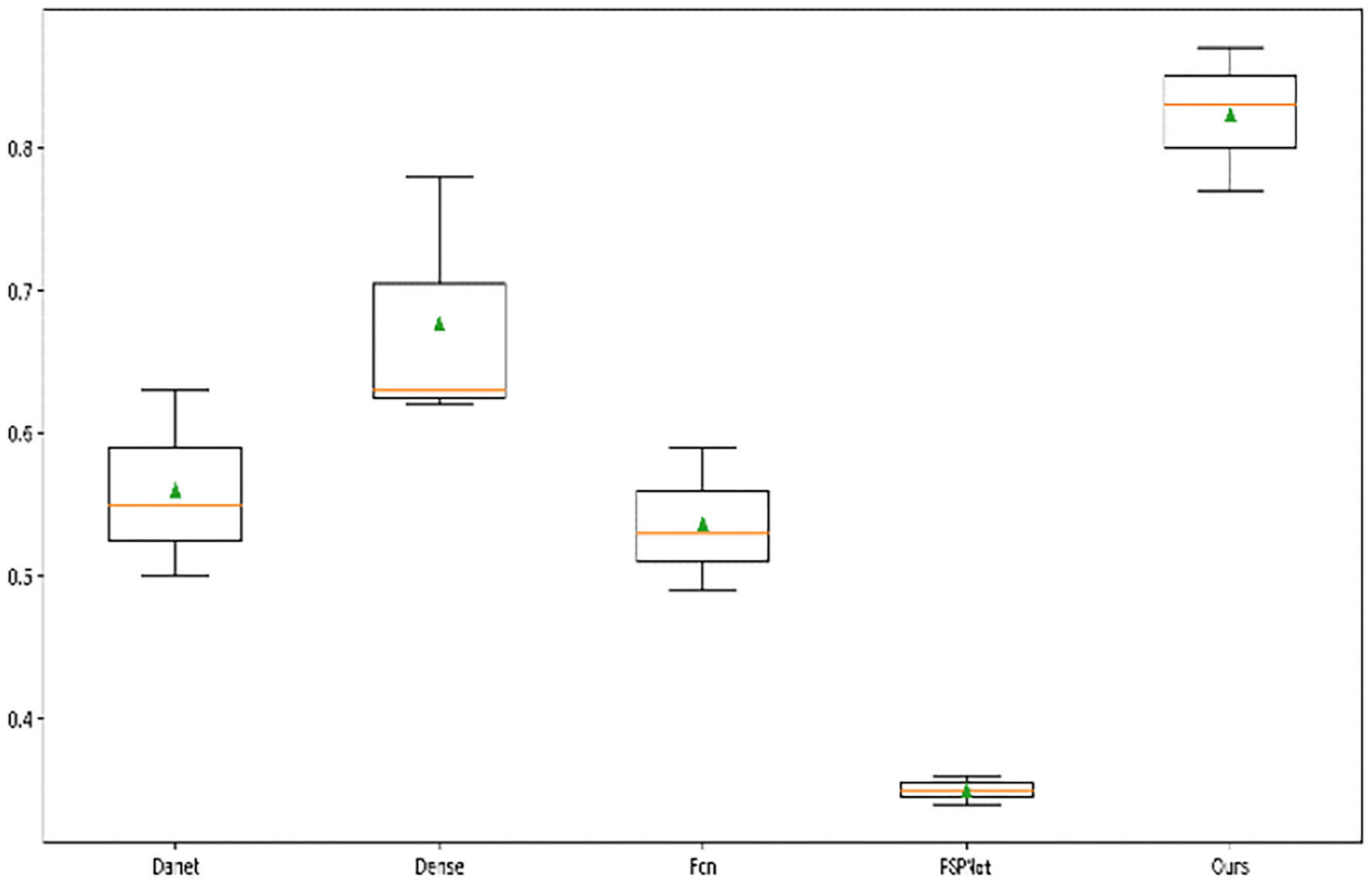
Box diagram of different model indexes.

In addition, we drew a box diagram (Figure 8) to demonstrate the overall and critical features of the data. There were Danet, Dence, Fcn, PSPNet, and the MIoU indicators of segmentation network under different backbones, and all model indexes were drawn into a box diagram.

It can be seen from the five models that the median accuracy of PSPNet algorithm was only 30%, but the lower quartile accuracy of Danet, Dense, and FCN were higher than 50%. The median of the algorithm was higher than 80%, displaying that our attention-based strategy had better performance in most samples and showed the robustness of the algorithm. Although other algorithms have achieved excellent performance on many datasets, the overall recognition effect of TD lesions is not satisfying. It is believed that the reason is that TD lesion is a small target and easily confused with other surrounding organs, especially the esophagus area. Even many experienced doctors often confuse or fail to identify the lesion, which results in obstacles to later diagnosis of diseases.

## Conclusion and Future Work

This work presented a new method of TD lesion recognition based on CT image semantic segmentation. Most of the previous studies focused on case reports, rather than using the deep learning method for segmentation to reduce the workload of doctors. In terms of that, we conducted pixel-level segmentation for the lesion area of CT images (that is, semantic segmentation) instead of traditional classification and target detection tasks. Inspired by the great success of attentional mechanism in image segmentation, we adopted the dual-channel attention model to automatically identify TD lesions and different pretrained feature extraction networks, including VGG16, DENASPP121, and ResNet50 as the backbone of the encoder, to improve the prediction performance of the network. Also, the position-coding and contour-coding module were redesigned with the integration of spatial and positional attention into the network, improving the identification of lesion position with coarse and fine-grained information. It has been proved that the segmentation network with Resnet, Mobilnetv2, Drn16, and Xcepotionv2 as the backbone of the encoder and the proposed coding module can effectively restore the details of the lesion area, superior to other popular semantic segmentation models, and novel algorithms based on U-net deformation. In other words, the value of MioU was 0.87, fully explaining that the semantic segmentation method is conducive to identifying TD lesions.

However, it should be noted that the research still has some limitations. First of all, because the above CT image resolution was low even with occlusions somewhere, it will be helpful to improve the network's performance once they are improved. Furthermore, more complex CT data should be introduced to enhance the robustness of the model. Ultimately, other ignored diseases like TD deserve further study so that deep learning can be applied to disease diagnosis and be integrated into a network with stronger generalization and migration ability. Once similar lesions with fixed positions and small targets are met, we can segment the network to obtain the needed results. Moreover, we will design an application to assist in the diagnosis of TD, with the aim of improving the diagnosis efficiency of medical practitioners.

## Data Availability Statement

The original contributions presented in the study are included in the article/supplementary material, further inquiries can be directed to the corresponding author.

## Ethics Statement

Written informed consent was obtained from the individual for the publication of any potentially identifiable images or data included in this article. The studies involving human participants were reviewed and approved by the Fengxian People's Hospital. The participants provided their written informed consent to participate in this study.

## Author Contributions

CD: resources and project administration. SG: overall experimental idea design and article editing. MZ: writing—original draft preparation and verification. All authors contributed to the article and approved the submitted version.

## Funding

This work was supported by Medical Research Project of Jiangsu Provincial Health Department (YG201419).

## Conflict of Interest

The authors declare that the research was conducted in the absence of any commercial or financial relationships that could be construed as a potential conflict of interest.

## Publisher's Note

All claims expressed in this article are solely those of the authors and do not necessarily represent those of their affiliated organizations, or those of the publisher, the editors and the reviewers. Any product that may be evaluated in this article, or claim that may be made by its manufacturer, is not guaranteed or endorsed by the publisher.
